# Diagnostic accuracy of symptoms compared to endoscopy, biopsy and bile reflux index in detecting reflux-related abnormalities at one year after OAGB

**DOI:** 10.1007/s00423-025-03748-y

**Published:** 2025-06-09

**Authors:** Mohamed Hany, Kareem El-Ansari, Walid El Ansari

**Affiliations:** 1 Department of Surgery, Medical Research Institute, Alexandria University, Alexandria, Egypt; 2 Madina Women’s Hospital, Alexandria University, Alexandria, Egypt; 3 Faculty of Medicine, St. George’s University, Saint George’s, Grenada; 4 College of Medicine, Ajman University, Ajman, United Arab Emirates

**Keywords:** Reflux, OAGB, Endoscopy, Diagnostic accuracy, Biopsy, GERD, Symptoms

## Abstract

**Background:**

The diagnostic accuracy of clinical symptoms in detecting reflux-related abnormalities after One anastomosis gastric Bypass (OAGB) remains unclear. This study evaluates the diagnostic performance of reflux symptoms compared to upper endoscopy (UE), biopsy, and bile reflux index (BRI) findings at one-year post-OAGB.

**Methods:**

A retrospective analysis was conducted on 150 consecutive patients who underwent OAGB between November 2017 and June 2018 and had no preoperative reflux symptoms. At one year postoperatively, patients completed the Gastroesophageal Reflux Disease Questionnaire (GerdQ) for symptom assessment. UE, histopathological biopsy, and BRI calculations were performed. The diagnostic accuracy of symptoms was evaluated against UE, biopsy, and BRI findings using sensitivity, specificity, positive predictive value (PPV), negative predictive value (NPV), and the area under the receiver operating characteristic curve (AUROC).

**Results:**

Among 144 patients analyzed, 25.7% reported GERD symptoms, while abnormal findings were observed in 62.5% (UE), 65.3% (biopsy), and 19.4% (BRI). Symptoms demonstrated high specificity and PPV (100%) in predicting UE and biopsy abnormalities but had low sensitivity (41.1% for UE, 39.4% for biopsy) and moderate NPVs (50.5% and 46.7%, respectively), indicating a risk of false negatives. The AUROC values were 0.71 (UE) and 0.70 (biopsy), reflecting moderate diagnostic discrimination. For BRI, symptom presence had 88.8% specificity and 64.9% PPV, but symptom absence correlated with high sensitivity (85.7%) and excellent NPV (96.3%), yielding an AUC of 0.87. Notably, 95.8% of symptomatic patients with abnormal BRI exhibited anastomotic site abnormalities, and 95.7% of patients with anastomotic pathology had concurrent distal esophageal and gastric pouch abnormalities.

**Conclusions:**

Symptoms may serve as a predictor of reflux-related abnormalities on UE or biopsy, but their absence is unreliable in ruling out such abnormalities. While symptoms effectively forecast abnormal BRI in high-prevalence settings, their diagnostic utility remains limited. Further research is warranted to assess long-term diagnostic accuracy and refine post-OAGB reflux assessment protocols.

## Introduction

Metabolic and bariatric surgery (MBS) is widely recognized as the most effective and durable intervention for severe obesity [1]. Within this domain, various surgical techniques are gaining traction [2–4], with One Anastomosis Gastric Bypass (OAGB) emerging as a favored approach due to its significant efficacy in achieving weight loss and improving obesity-related comorbidities [5–7]. OAGB has received validation from the American Society for Metabolic and Bariatric Surgery [8] and the International Federation for the Surgery of Obesity and Metabolic Disorders [9, 10], leading to its increased adoption worldwide.

A contentious issue in the field remains the prevalence of gastroesophageal reflux disease (GERD) following OAGB, with conflicting reports on its incidence. Bile reflux (BR) as part of GERD has been documented to occur in 0–4% of patients across high-volume studies [5, 11–17]. However, comparative analyses between sleeve gastrectomy and OAGB indicate that by year three, the incidence of de novo GERD was significantly higher in sleeve gastrectomy patients [7]. A pooled analysis of 70 research studies found that the rate of new-onset reflux post-OAGB stands at approximately 6%, comparable to rates observed in sleeve gastrectomy cases [18]. This discrepancy in findings may be attributed to a diverse array of reflux diagnostic methodologies and their varying correlations with the symptomatic presentation.

The diagnosis of GERD typically utilizes a combination of symptom assessment, ambulatory esophageal pH monitoring, endoscopy, and anti-secretory therapy response [19], among other diagnostic tools. However, these approaches often possess inherent limitations, including patient discomfort, impact on daily living, variable sensitivity, and associated costs [19]. Currently, no definitive ‘gold standard’ exists for GERD diagnosis; however, upper endoscopy (UE) and biopsy are among the most frequently employed techniques. Notably, the diagnostic efficacy of these modalities has not been thoroughly validated after OAGB.

Moreover, the correlation between symptomatology and UE or biopsy outcomes remains poorly characterized. Following OAGB, the presence or absence symptom does not reliably serve as a definitive GERD diagnostic tool, as studies have suggested a lack of correlation between symptom severity and the extent of esophageal damage [20–22]. Thus, to achieve a more conclusive diagnosis, there is a growing consensus for the incorporation of UE and biopsy [23, 24].

An additional critical factor is the bile reflux index (BRI), an established histological measure used to evaluate bile reflux-related gastric mucosa injury. Elevated BRI values in GERD patients support the hypothesis that duodeno-gastroesophageal reflux contributes to GERD pathogenesis [25]. A study of esophagitis and bile reflux gastritis used BRI and found milder esophagitis in patients with gastric surgery exposed mainly to bile than in those with intact stomachs exposed to both gastric acid and bile [26]. Others reported that for the reflux of bile into the esophagus, the BRI, calculated by an experienced pathologist, represents a reliable tool for detecting bile reflux [25]. Similarly, a comparison of the bile reflux frequency in OAGB versus Roux-en-Y gastric bypass employed BRI as one of the indicators [27].

The existing literature reveals significant gaps regarding the diagnostic validation of symptoms to endoscopic and histopathologic findings after OAGB. Although some studies have assessed the utility of combined symptomatology and UE in reflux detection [15, 28, 29], no comprehensive evaluation comparing these parameters has been conducted to ascertain their associations or diagnostic accuracy. Additionally, the relationship between BRI and clinical symptoms or diagnostic findings remains unexamined, to the best of the authors’ knowledge.

The current study aims to bridge these knowledge gaps by evaluating the presence of GERD symptoms alongside UE, biopsy, and BRI alterations in a cohort of 150 patients after OAGB. The specific objectives focus on four interrelated research questions, outlined in Table 1, which will elucidate the interplay between symptoms and diagnostic outcomes one year after the procedure.

**Table 1:** Research questions and specific objectives of current study and their outcome

Research question	Objective
1. Proportion of patients with GERD symptoms, abnormal UE, biopsy and/or abnormal BRI findings?	Prevalence of GERD symptoms, abnormal UE, biopsy, and BRI findings
2. Diagnostic accuracy of symptoms compared to UE, biopsy and BRI findings?	Five indices: sensitivity, specificity, PPV, NPV, AUROC of symptoms vs. each of UE, biopsy and BRI
3. Implications of symptoms or lack thereof on forecasting UE, biopsy and BRI findings?	Utility of:
	a) Presence of symptoms in forecasting UE, biopsy, and BRI abnormalities
	b) Lack of symptoms in excluding UE, biopsy, and BRI abnormalities
4. Relationship between symptoms, BRI, and biopsy findings?	Distribution of diagnostic outcomes, characterized by symptoms, BRI, and biopsy

*BRI* bile reflux index, *UE* upper endoscopy, *PPV* positive predictive value, *NPV* negative predictive value, *AUROC* area under the receiver operating characteristic curve, Analysis by overall abnormalities (i.e., regardless of anastomotic site)

## Materials and methods

### Study design and ethics

The current study is a retrospective analysis of prospectively collected data on OAGB conducted at the Medical Research Institute from November 2017 to June 2018. This study received approval from our institution’s Ethics Committee (approval # IORG0008812; E/C.S/N.R4/2017), and all participating patients provided written informed consent.

### Inclusion and exclusion criteria

Adult patients undergoing OAGB during the specified timeframe were included, with criteria specifying a BMI > 40 kg/m² or > 35 kg/m² with comorbid conditions [30]. Exclusion criteria encompassed individuals with prior MBS, preoperative GERD symptoms, or a history of ulcers or Barrett’s esophagus. Additionally, those on regular proton pump inhibitors (PPIs) or H2 receptor antagonists were instructed to discontinue such medication two weeks before UE to mitigate its potential effects on reflux symptomatology [31–33].

### Participants

Our institution provides OAGB services to both local and international patients. Out of 264 consecutive OAGB cases during the study period, 114 were excluded for not meeting the inclusion criteria: 50 returned overseas, 24 were over 60 years old, 6 had large hiatal hernias (> 5 cm), 16 presented with GERD, and 18 had previous metabolic/bariatric surgeries or abdominal explorations. Consequently, 150 patients consented and were incorporated into the study. By the one-year follow-up, six patients were lost to follow-up, resulting in a cohort of 144 patients for analysis.

### Data collection

Data was collected one year after OAGB to evaluate early postoperative reflux, aligning with previous studies that examined UE and histopathological changes as early as six months to two years post-surgery [23, 24]. Three primary data sets were collected: clinical symptoms, results from UE, and histopathological biopsy analysis.

### GERD assessment via GerdQ

Patients underwent evaluation for GERD symptoms through the Gastroesophageal Reflux Disease Questionnaire (GerdQ), a validated diagnostic tool comprising six items with a scoring range of 0–18 [34]. Each item evaluates symptoms including heartburn, regurgitation, epigastric discomfort, nausea, sleep disturbances due to heartburn, and the use of over-the-counter antacids, where the maximum score per question is 3. A cutoff score of 8 indicates a likelihood of GERD with 65% sensitivity and 71% specificity, and scores ≥ 8 points indicate an 80% chance of having GERD [11, 34–36]. Notably, GerdQ has been utilized in prior studies evaluating gastroesophageal reflux in MBS populations [33, 37]. The questionnaire was administered without translation into Arabic by a bilingual researcher, trained in the nuances of the questions and administration techniques to mitigate any bias during patients’ responses. Before questionnaire administration, individuals who were using PPIs or H2 blockers were instructed to discontinue these medications for two weeks [33, 38].

### Upper gastrointestinal endoscopy protocol

UE was conducted under conscious sedation, with patients fasting from solid food for 6–8 h before the procedure. An experienced endoscopist utilized high-definition endoscopy to assess the distal esophagus, gastric pouch, and anastomotic site, systematically collecting biopsy samples for histological analysis from these levels. A standardized documentation template was utilized to classify findings at each anatomical site as normal or abnormal, including evaluation for hiatus hernias, incompetent cardia, mucosal abnormalities in the distal esophagus; erosive or non-erosive gastritis in the gastric pouch; and mucosal hyperemia or ulcers at the anastomotic site [39]. In adherence to NICE guidelines, participants refrained from PPI or H2 receptor antagonist therapy for at least two weeks before endoscopy to avoid false-negative results [40].

### Biopsy (histopathology) protocol

A standardized protocol was employed to conduct multiple biopsies (3 passes, yielding 6 specimens) from three distinct anatomic sites. All samples were embedded in paraffin after fixation in 10% buffered formalin, sectioned at 4 μm, and subsequently stained with hematoxylin-eosin and modified Giemsa stain. An experienced pathologist examined the specimens microscopically while remaining blinded to the patient’s clinical presentation and UE findings. Observations included the presence or absence of bile in the esophagus and gastric pouch.

The biliary reflux index (BRI) was calculated to assess duodeno-gastric reflux, which incorporated parameters such as edema in the lamina propria (E), intestinal metaplasia (IM), chronic inflammation (CI), and gastric H. pylori (Hp) colonization. Each histological parameter was graded on a scale of 0 to 3 (absent to marked). The BRI value was derived from the formula: BRI = (7 × E) + (3 × IM) + (4 × CI) - (6 × Hp). A BRI value exceeding 14 indicated the presence of duodeno-gastric reflux, defined as bile acid levels > 1 mmol/L, which represents the upper limit of physiological reflux [41, 42].

### Surgical technique

All procedures were performed by a single experienced bariatric surgeon. The patient was positioned in modified lithotomy, and five trocars were inserted utilizing standard port placement. Initial dissection of the lesser omentum was conducted below the crow’s foot to facilitate stapler passage. A long gastric pouch was constructed using a linear stapler and reloads over a 40-Fr bougie, with the first reload applied transversely below the incisura angularis, followed by vertical reloads directed toward the angle of His, ensuring dissection of the angle of His and posterior gastric adhesions.

Biliopancreatic limb lengths were determined based on BMI: 200 cm for a BMI of ≥ 50 kg/m² and 150 cm for a BMI < 50 kg/m², maintaining a minimum common limb length of 300 cm. Blue reloads were employed during gastrojejunostomy construction, with stapling defects closed using continuous sutures of 3/0 V-Loc 180 (Covidien). The gastric pouch was verified to be at least 15 cm in length above the gastrojejunostomy.

The staple lines were further reinforced with invaginating sutures of the same barbed materials. In cases with preoperatively diagnosed hiatal hernia, the crural repair was performed using 2/0 V-Loc nonabsorbable sutures (Covidien). Concomitant cholecystectomy was executed for cases with diagnosed calcular cholecystitis. An intraoperative methylene blue leak test was routinely conducted, and a tube drain was placed in the left subphrenic space.

All patients underwent preoperative H. pylori testing, followed by eradication therapy if the results were positive. Additionally, patients were instructed to abstain from smoking and alcohol for 4 to 6 weeks before surgery [33].

### Statistical analysis

Statistical analyses utilized R software version 4.2.2 [43]. Descriptive statistics summarized continuous data as means and standard deviations (M ± SD) and categorical data as frequencies and percentages. The diagnostic accuracy of clinical symptoms relative to UE findings, biopsies, and BRI was assessed through metrics such as sensitivity, specificity, positive predictive value (PPV), negative predictive value (NPV), and area under the receiver operating characteristic curve (AUROC). The 95% confidence interval provided an estimate of precision for these metrics. Following established criteria, AUROC interpretation was classified as follows: 0.5 = no discrimination; >0.5 to < 0.7 = poor; 0.7 to < 0.8 = acceptable; 0.8 to < 0.9 = excellent; and AUROC ≥ 0.9 = outstanding discrimination [44].

## Results

### Characteristics of the sample: prevalence of symptoms, and abnormal UE, biopsy and BRI

Table 2 outlines the preoperative characteristics of the 150 patients included in this study. Mean age was 34.7 years (range 18–60), and 75.3% were females. Mean BMI was 43.8 kg/m^2^. Table 3 shows the findings at one year post-OAGB. Although 25.7% of patients were symptomatic (GerdQ score ≥ 8), however, 62.5% and 65.3% exhibited UE and biopsy abnormalities, respectively, and 19.4% had BRI that was in the abnormal range.

**Table 2:** Preoperative characteristics of the sample (*N* = 150)

Characteristic	Value *n* (%)
Age (years)	
M ± SD	34.7 ± 11.4
Median (Range)	32.5 (18–60)
Sex (Female)	113 (75.3)
Smoking (Yes)	19 (12.7)
BMI (kg/m^2^)	
M ± SD	43.8 ± 3.2
Median (Range)	43.42 (36.0–54.1)

Cell values represent frequency (percent) unless otherwise stated; M ± SD: mean ± standard deviation

**Table 3:** GerdQ score, and endoscopic and biopsy findings at one year after OAGB (*N* = 144)

Finding	Value
GerdQ score	
Symptomatic (≥ 8)	37(25.7)
Asymptomatic (< 8)	107(74.3)
Endoscopic	
Normal	54(37.5)
Abnormal^*a*^	90(62.5)
Biopsy	
Normal	50(34.7)
Abnormal^*b*^	94(65.3)
Bile Reflux Index	
Normal (< 14)	116(80.6)
Abnormal (≥ 14)	28(19.4)

Cell values represent frequency (%); ^*a*^overall includes hiatus hernia, incompetent cardia, mucosal abnormality, non-erosive gastritis, erosive gastritis, ulcer; ^*b*^overall includes chronic esophagitis, chronic gastritis, ulcer, H. Pylori

### Diagnostic accuracy: symptoms vs. overall UE, overall biopsy, and BRI

**Fig. 1:**
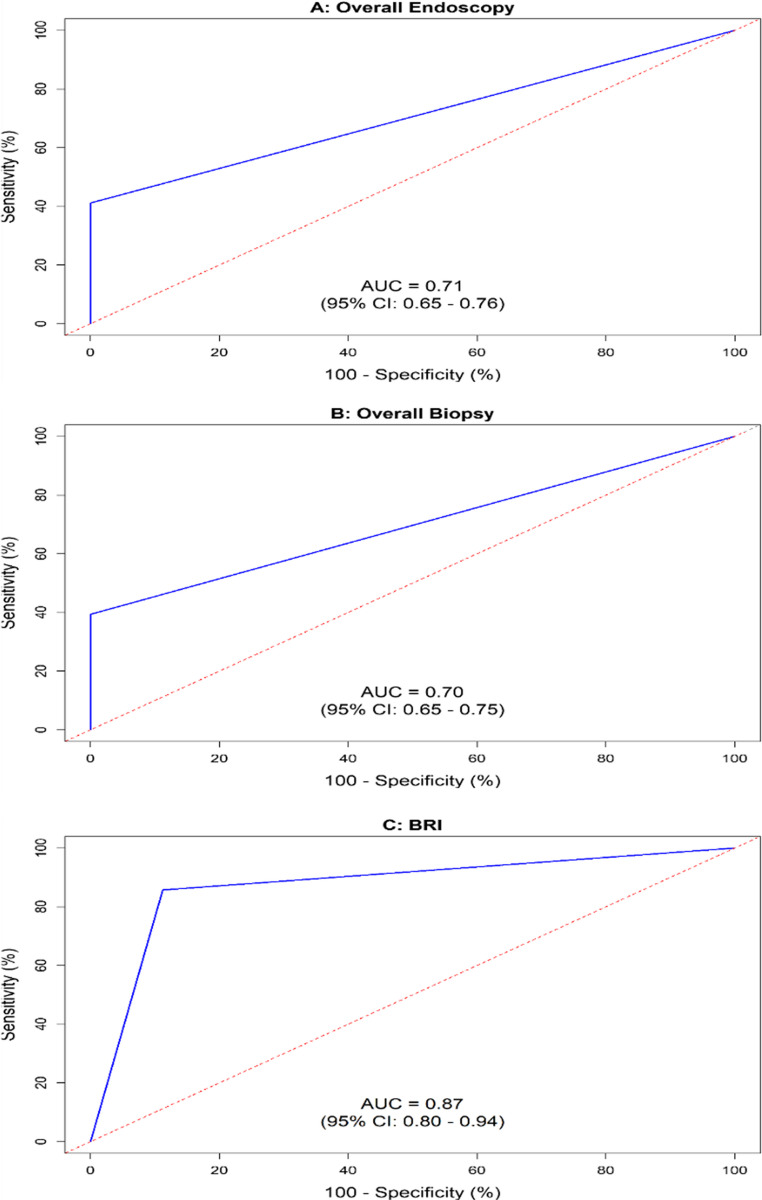
AUROC curves of symptoms compared to overall endoscopic findings (panel **A**), overall biopsy findings (panel **B**) and to bile reflux index (panel **C**) one year after OAGB

Table 4; Fig. 1 show the diagnostic accuracy of symptoms compared to overall UE, overall biopsy and BRI findings at one year. Symptoms demonstrated 100% specificity and PPV in forecasting UE and biopsy abnormalities. Despite this, sensitivity of symptoms was low for both methods (41.1%, 39.4%), resulting in NPVs of 50.5% and 46.7%. Figure 1 shows that AUROC values of symptoms were just above the threshold for acceptable discriminative ability (0.71, 0.70). In contrast, Table 4 depicts that symptoms displayed high sensitivity (85.7%) and specificity (88.8%) in detecting abnormal BRI, with moderate PPV (64.9%) and high NPV (96.3%), achieving a superior AUROC of 0.87 (Fig. 1).

**Table 4:** Diagnostic accuracy: symptoms compared to overall endoscopy, overall biopsy, and bile reflux index one year after OAGB

Modality		GerdQ symptom score		Accuracy indices				
		Symptomatic (≥ 8)	Asymptomatic (< 8)	Sensitivity	Specificity	PPV	NPV	AUROC
		*n* = 37	*n* = 107	95% CI	95% CI	95% CI	95% CI	
Overall endoscopy	Abnormal	37(25.7)	53(36.8)	41.1(30.4–51.6)	100(100–100)	100(100–100)	50.5(40.4–59.8)	0.71(0.65–0.76)
	Normal	0(0.0)	54(37.5)					
Overall biopsy	Abnormal	37(25.7)	50(34.7)	39.4(28.8–49.3)	100(100–100)	100(100–100)	46.7(37.2–56.5)	0.70(0.65–0.75)
	Normal	0(0.0)	57(39.6)					
BRI	Abnormal (≥ 14)	24(16.7)	4(2.8)	85.7(65.4–95.8)	88.8(81.6–93.8)	64.9(48.3–79.0)	96.3(90.5–99.0)	0.87(0.80–0.94)
	Normal (< 14)	13(9.0)	103(71.5)					

Cell values represent n (%); values for accuracy indices represented as mean (95% Confidence interval)

*CI* Confidence interval, *PPV* positive predictive value, *NPV* negative predictive value, *AUROC* area under the receiver operating characteristic curve, *BRI* bile reflux index

### Diagnostic accuracy of symptoms: implications

**Fig. 2:**
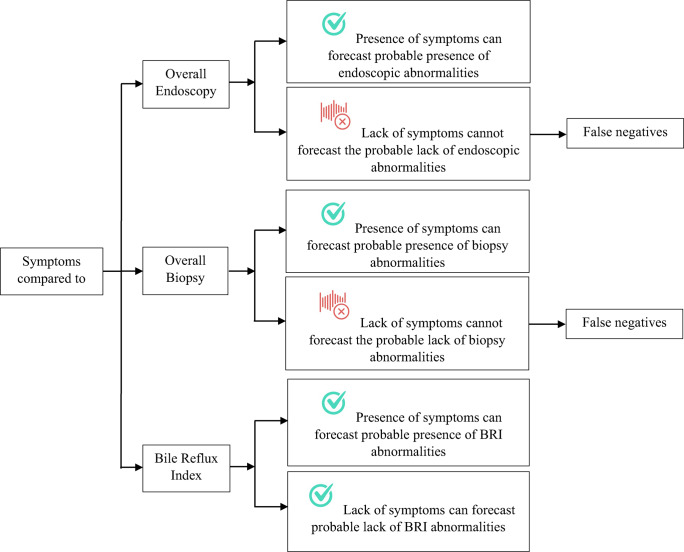
Utility of symptoms in forecasting probable endoscopic (UE), biopsy, and BRI abnormalities

Table 5 summarize the utility of presence of symptoms in forecasting UE, biopsy, and BRI abnormalities, and its absence in excluding such abnormalities. As for UE and biopsy, symptoms can be used to forecast probable UE and/or biopsy abnormalities; and lack of symptoms cannot be used to exclude probable UE and/or biopsy abnormalities as it could generate false negatives. Pertaining to BRI, symptoms can be used to forecast abnormal BRI only when prevalence of disease is high; but lack of symptoms can be used to exclude abnormal BRI. Figure 2 depicts the potential utility of symptoms or lack thereof in forecasting probable UE, biopsy, and BRI abnormalities or lack thereof.

**Table 5:** Diagnostic accuracy of symptoms compared to endoscopy, biopsy, and BRI one year after OAGB and their implications

Accuracy indices	Value	Comment
Symptoms vs overall endoscopy		
Specificity, PPV	High specificity + PPV (both 100%)	Effective for forecasting probable abnormalities
Sensitivity, NPV	Low sensitivity (41.1%) + NPV (50.5%)	Not reliable for forecasting probable lack of abnormalities, as many true abnormalities may be missed (false negatives)
AUROC	0.71	Fair overall discriminative ability
Symptoms vs overall biopsy		
Specificity, PPV	High specificity + PPV (both 100%)	Effective for forecasting probable abnormalities
Sensitivity, NPV	Low sensitivity (39.4%) + NPV (46.7%)	Not reliable for forecasting probable lack of abnormalities, as many true abnormalities may be missed (false negatives)
AUROC	0.70	Fair overall discriminative ability
Symptoms vs overall BRI		
Specificity, PPV	Good specificity (88.8%) + fair PPV (64.9%)	Good for forecasting probable abnormalities; but not at low prevalence of abnormalities as it could generate false positives
Sensitivity, NPV	Good sensitivity + NPV (85.7%, 96.3%)	Good for forecasting probable lack of abnormalities
AUROC	0.87	Good overall discriminative ability

*UE* upper endoscopy, *PPV* positive predictive value, *NPV* negative predictive value, *AUROC* area under the receiver operating characteristic curve, *BRI* bile reflux index

### Mapping of symptoms, characterized by BRI and biopsy findings one year after OAGB

**Fig. 3:**
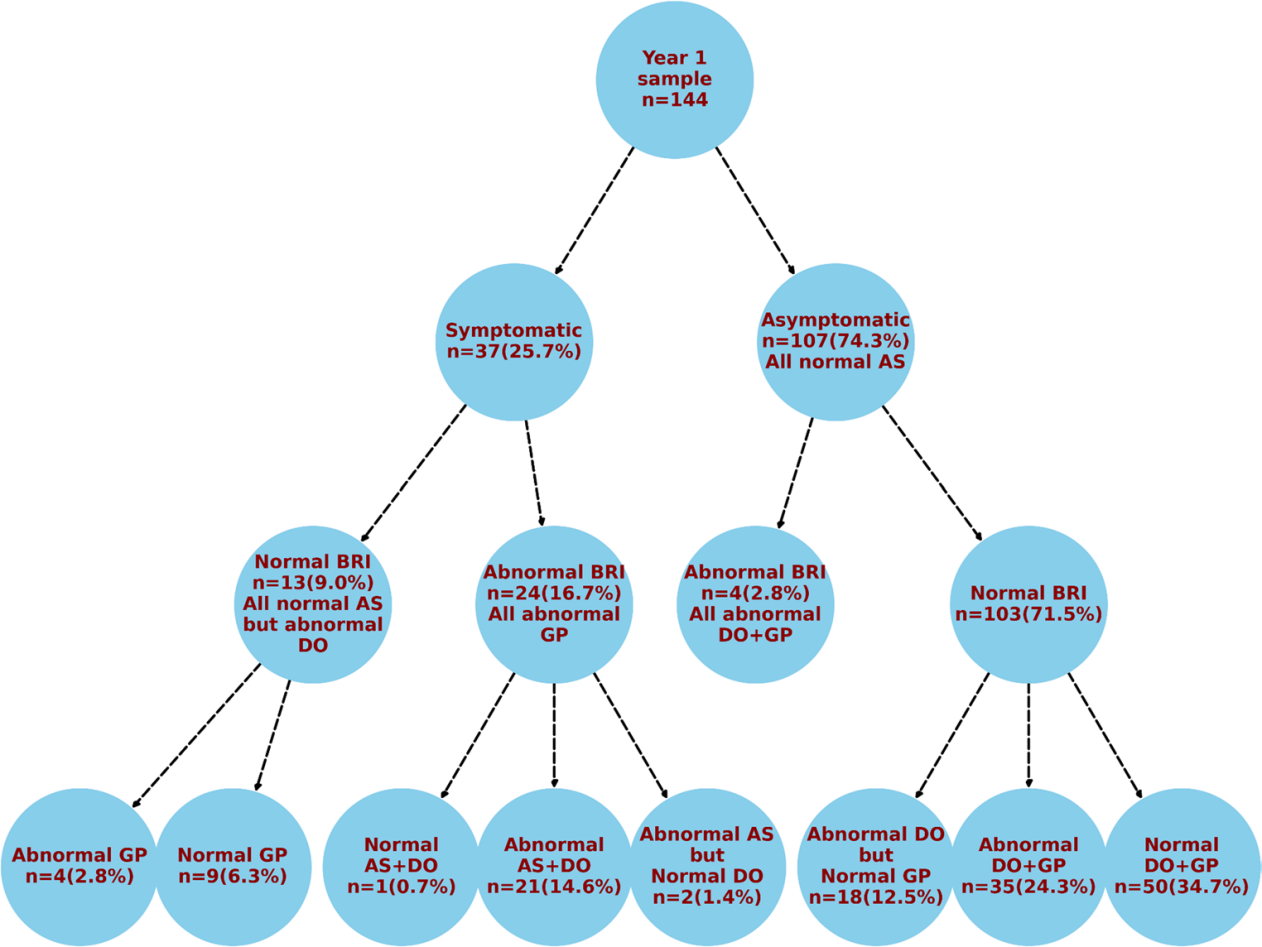
Diagnostic outcomes at one year after OAGB, categorized by GerdQ symptom status, BRI and biopsy (*N* = 144). BRI: Bile Reflux Index; DO: distal esophagus; GP: gastric pouch; AS: anastomotic site

Figure 3 shows the distribution of outcomes a year after OAGB, based on GerdQ symptom status, BRI, and biopsy results from the three anatomic sites. Anastomotic site was consistently normal in all asymptomatic patients irrespective of BRI status, and among all symptomatic patients with normal BRI. The findings suggested a link between symptomatic bile reflux and anastomotic site micro-pathology, as nearly all (95.8%) symptomatic patients with abnormal BRI exhibited concurrent abnormalities at the anastomotic site (87.5% with abnormal gastric pouch + distal esophagus; 8.3% with abnormal gastric pouch only). When anastomotic site abnormalities were present, the extent of pathological involvement appeared to extend beyond it, as 95.7% (22 out of 23) of patients with anastomotic site abnormality invariably had concomitant distal esophagus + gastric pouch abnormalities.

## Discussion

OAGB ranks as the third-most performed metabolic and bariatric surgery (MBS) according to the International Federation for the Surgery of Obesity and Metabolic Disorders (IFSO) registry, following sleeve gastrectomy and Roux-en-Y gastric bypass [1]. This procedure demonstrates remarkable metabolic outcomes, including remission of comorbid conditions and enhanced quality of life [7, 45, 46]. However, concerns about post-OAGB esophageal reflux persist. To date, there has been a lack of studies evaluating the diagnostic accuracy and clinical utility of reflux symptoms in comparison to established modalities such as UE, biopsy, and bile reflux index (BRI) following OAGB.

The present study contributes valuable insights into the complex relationships between post-OAGB reflux symptoms and the findings from UE, biopsy, and BRI. Our results indicate that the diagnostic accuracy of reflux symptoms varies when compared to UE, biopsy, and BRI, highlighting the nuances in utilizing symptoms as a first-line approach for diagnosing potential abnormalities. One year post-OAGB, the diagnostic accuracies of reflux symptoms aligned closely with overall findings from UE and biopsy. The presence of symptoms exhibited robust specificity and PPV, indicating its utility in predicting potential abnormalities in UE or biopsy. Conversely, the low sensitivity and NPV implied that the absence of symptoms was not reliable for ruling out abnormalities, posing a risk for false negatives.

In terms of BRI, the presence of reflux symptoms showed strong sensitivity (85.7%) and specificity (88.8%) in predicting potential BRI abnormalities. However, the moderate PPV (64.9%) along with a high NPV (96.3%) suggests that this predictive capacity is most effective in high-prevalence scenarios, as the absence of symptoms could indicate an absence of abnormal BRI.

Regarding the primary and secondary objectives of our study, we observed a prevalence of 25.7% for gastroesophageal reflux disease (GERD) symptoms, alongside 62.5% abnormal UE, 65.3% abnormal biopsy results, and 19.4% abnormal BRI findings. It is important to note that no previous studies have directly compared the diagnostic accuracy of reflux symptoms against the outcomes of UE, biopsy, and BRI in this context [15, 28, 29, 39]. Some prior research has attempted to combine clinical symptoms with UE for reflux detection but did not adequately evaluate their relative utility or the correlation between symptomatology and macroscopic abnormalities observed during UE.

Among the bariatric population, the relationship between reflux symptoms and their correlation with actual macro/microscopic abnormalities remains contentious. Previous investigations have similarly pointed out the insufficiency of sensitivity and specificity data related to reflux diagnostic techniques, complicating direct comparisons along with the additional layer of analysis presented in our study involving symptom evaluation against conventional reflux detection methods [47].

Regarding our third objective, the present series indicates that the presence of symptoms one year following OAGB may serve as a useful prognostic tool for predicting the likelihood of UE and biopsy abnormalities. Our findings align with existing literature, which demonstrates that patients experiencing upper gastrointestinal symptoms within three months post-gastric bypass surgery are at an increased risk for abnormal UE findings [48].

Notably, a systematic review and various studies have revealed that a majority of UE-diagnosed ulcers following gastric bypass presented with symptoms, with asymptomatic cases constituting only 17–28% [49–51]. After one month of surgery, approximately 75% of patients in Chile with MU exhibited symptoms [50]; further, a meta-analysis of post-OAGB MUs confirmed that most patients (54 out of 65) with UE-diagnosed MUs reported symptoms [49]. However, these studies did not assess the diagnostic accuracy associated with symptoms [49, 50].

There is existing literature exploring symptomatology in conjunction with different diagnostic modalities, which cannot be directly compared to our findings. For instance, among 46 patients with epigastric pain, scintigraphic imaging using fasting hourly bile acid aspiration confirmed duodenogastroesophageal reflux in 80.4% of cases. However, this study’s context—conducted outside the OAGB setting, alongside limitations such as a small sample size and the absence of an asymptomatic control group—restricts its applicability [52]. Other comparative studies did not incorporate symptoms into their analysis; for example, hepatobiliary iminodiacetic acid (HIDA) scintigraphy demonstrated superior sensitivity and specificity compared to UE and gastric fluid aspiration [53].

Our findings yield two significant insights. First, while we established that the presence of symptoms may serve as a provisional indicator for UE and biopsy abnormalities, these symptoms do not specify the nature or type of the abnormalities. Thus, symptoms act as a critical signaling mechanism, suggesting the potential existence of UE/histopathological lesions that necessitate further investigation without accurately characterizing the specific abnormalities present.

The second insight pertains to the anatomical location of the UE/biopsy abnormalities. The high specificity and PPV observed with symptoms indicate a correlation with UE/biopsy abnormalities irrespective of anatomical site (distal esophagus, gastric pouch, or anastomotic site). This analysis focused on an overarching assessment of UE/biopsy abnormalities without distinguishing between their locations.

In summary, our study found a concordance between the presence of symptoms and UE/biopsy abnormalities. However, previous studies reported discordant findings regarding symptomatology and histological changes in the esophagus, cardiac region, and gastric pouch among post-OAGB patients [11, 27, 47]. This apparent contrast may stem more from methodological differences than from true discrepancies, likely influenced by the focus of our study compared to others. We conducted a comprehensive diagnostic accuracy analysis aimed at assessing overall abnormalities, whereas previous research employed a more generalized approach that did not include diagnostic accuracy indices but rather, provided a broad characterization of symptom-histological agreement relative to specific anatomical locations [11, 27, 47].

This tradeoff—balancing the depth of diagnostic accuracy against the specificity of anatomical locations—can account for the divergent results between our findings and those of others. Importantly, our analysis equips clinicians with clearer indications regarding the utility of symptoms for ruling in or out overall reflux-related UE or biopsy abnormalities, a nuance not explicitly addressed in prior studies.

In the current study, the absence of symptoms cannot reliably predict the absence of UE or biopsy abnormalities due to the low sensitivity and negative predictive value (NPV) of such assessments, which can result in false negatives. This necessitates a reconsideration of the interpretation of asymptomatic patients as potentially having underlying diseases. For example, in cases following post-OAGB surgery, studies typically focus UE evaluations on symptomatic patients, risking an underestimation of the actual incidence of MU [13, 17, 54]. Notably, one month after surgery, 28% of patients with MU remained asymptomatic [50], and at the one-year follow-up, 9.5% of 42 post-OAGB patients undergoing UE had MU, all of whom were asymptomatic [55]. A meta-analysis on post-OAGB MU further corroborated this, revealing that 11 out of 65 patients diagnosed with MU via UE were asymptomatic [49].

False-negative findings must be avoided as they can lead to serious consequences, including legal repercussions, diminished public trust, delayed diagnoses, and increased morbidity and mortality rates [56–58]. Our results align with this perspective, reinforcing the notion that asymptomatic status should not be used as a criterion to forgo UE or biopsy due to the aforementioned limitations in sensitivity and NPV.

Regarding our fourth objective, the data suggests that symptomatic presence, given the high specificity and sensitivity of BRI but only fair PPV, may indicate abnormal BRI findings predominantly in high-prevalence contexts. However, this strategy is generally inadvisable in scenarios where prevalence is low or unknown. While scant research exists on esophageal bile reflux in the context of OAGB procedures [11, 27, 39, 59–62], instances of bile reflux have rarely been documented in such cases [39, 59]. The lack of published literature regarding the correlation between GerdQ symptomatology, BRI, and biopsy outcomes one-year post-OAGB limits our ability to draw direct comparisons to our findings.

One study assessing esophageal bile reflux in 20 OAGB patients, which analyzed GerdQ symptoms, biliary scintigraphy, and UE, found no significant relationship between reported symptoms and positive reflux on scintigraphy [39]. Observations of symptomatic bile reflux demonstrated a correlation with micro-pathology at the anastomotic site, with nearly all symptomatic patients having abnormal findings at this location [63]. Furthermore, when abnormalities were noted at the anastomotic site, a notable majority (95.7%) exhibited concurrent pathological changes in the distal esophagus and gastric pouch [33]. The current study supports the call for further investigations involving larger cohorts to distinguish between new cases of gastroesophageal reflux disease (GERD) and bile reflux, employing objective assessments such as UE with biopsy.

Our findings also endorse the adoption of multi-modal investigative approaches for reflux. Previous literature suggested that integrating scintigraphy to diagnose bile reflux with UE for macro- and microscopic mucosal evaluation should be regarded as a gold standard for diagnostic exploration [47].

The present study is not without limitations. We did not delineate the spectrum of reflux symptoms, distinguish between endoscopic or histopathological specific lesion types, or categorize the lesion by anatomical sites (distal esophagus, gastric pouch, anastomotic site) involved in UE or histopathological assessments, which could have complicated the interpretability of our results. Consistent with previous research, we also did not differentiate between acid and bile reflux phenomena [5, 33, 64]. Previous findings indicate that symptomatic GERD often involves a convergence of biliary and acid reflux, complicating symptom-based differentiation [38]. We acknowledge that routine comprehensive immediate preoperative upper gastrointestinal endoscopy was not available for all patients, which may limit the ability for definitive findings prior to surgery. Future research should address these points, as well as including comparison groups that had undergone other MBS procedures e.g., Roux-en-Y gastric bypass. Despite these limitations, this study possesses considerable strengths. It is the first to evaluate 150 patients simultaneously for the diagnostic accuracy of symptoms against UE, biopsy findings, and BRI alterations one-year following OAGB, addressing four interrelated queries. Crucially, it utilized five indices to provide a comprehensive understanding of the relationships and their temporal variations, evaluating the role of symptom presence or absence in predicting UE or histopathological changes. Additionally, it offers a detailed breakdown of diagnostic outcomes after one-year post-OAGB, categorized by GerdQ symptom status, BRI, and biopsy results. Therefore, the diagnostic capacity of symptomatology relative to other objective measures warrants further exploration, particularly within the framework of post-OAGB patient cohorts.

## Conclusion

The diagnostic accuracy of clinical symptoms in relation to UE and biopsy findings exhibited notable similarities, diverging from those observed with the BRI. Consequently, the effectiveness of symptoms in confirming or excluding abnormalities detected by these modalities is distinct. The presence of symptoms serves as a useful predictor for identifying potential abnormalities in UE or biopsy; however, the absence of symptoms proved to be an unreliable indicator for the absence of findings in these modalities, leading to a significant rate of false negatives.

Conversely, while the presence of symptoms demonstrates strong sensitivity and specificity for anticipating BRI abnormalities, this correlation is only reliable in high-prevalence contexts, making it less advisable in general practice. Notably, the absence of symptoms may suggest a likely absence of abnormal findings in the BRI context. Future studies are necessary to further elucidate the relationships between symptoms and the diagnostic outcomes of UE, biopsy, and BRI.

## Data Availability

Data can be shared upon reasonable request and subject to agreement of the institution where the research was implemented.
